# Prognostic predictors of adrenocortical carcinoma: A single-center thirty-year experience

**DOI:** 10.3389/fendo.2023.1134643

**Published:** 2023-03-10

**Authors:** Li-Hsin Pan, Chueh-Chuan Yen, Chun-Jui Huang, Xin-Ning Ng, Liang-Yu Lin

**Affiliations:** ^1^ Division of Endocrinology and Metabolism, Department of Medicine, Taipei Veterans General Hospital, Taipei, Taiwan; ^2^ Faculty of Medicine, School of Medicine, National Yang Ming Chiao Tung University, Taipei, Taiwan; ^3^ Division of Clinical Research, Department of Medical Research, Taipei Veterans General Hospital, Taipei, Taiwan; ^4^ Division of Medical Oncology, Center for Immuno-oncology, Department of Oncology, Taipei Veterans General Hospital, Taipei, Taiwan; ^5^ Institute of Biopharmaceutical Sciences, College of Pharmaceutical Sciences, National Yang Ming Chiao Tung University, Taipei, Taiwan; ^6^ Division of Endocrinology and Metabolism, Department of Medicine, Dalin Tzu Chi Hospital, Buddhist Tzu Chi Foundation, Chiayi, Taiwan

**Keywords:** adrenocortical carcinoma, mitotane, overall survival (OS), progression-free survival (PFS), adrenal carcinomas

## Abstract

**Background:**

The prognosis of adrenocortical carcinoma (ACC) is poor but highly variable. The present study aimed to characterize patients with ACC at a single center in Taiwan and to determine the prognostic predictors of overall and progression-free survival.

**Methods:**

Medical records of patients, who were diagnosed with ACC at Taipei Veterans General Hospital between January 1992 and June 2021, were reviewed. Patient demographics, tumor characteristics, and subsequent treatment were analyzed with regard to overall survival and progression-free survival using Kaplan-Meier methods and a Cox regression model.

**Results:**

Sixty-seven patients were included. Females (65.7%) were more susceptible to ACC, with a younger onset and active hormonal secretion. One-half of the patients exhibited distant metastases at the time of diagnosis. The European Network for the Study of Adrenal Tumours (ENSAT) stage (hazard ratio [HR] 3.60 [95% confidence interval (CI) 1.25–10.38]; p=0.018), large vessel invasion (HR 5.19 [95% CI 1.75–15.37]; p=0.003), and mitotane use (HR 0.27 [95% CI 0.11–0.70]; p=0.007) were significantly associated with overall survival (OS). There was no single factor independently associated with progression-free survival.

**Conclusion:**

ENSAT stage had a substantial impact on overall survival though there was no difference in OS between patients with stage II and stage III ACC. Large vessel invasion portended poor prognosis and influenced OS significantly. Moreover, mitotane only improved clinical outcomes of patients with stage IV disease.

## Introduction

Adrenocortical carcinoma (ACC) is a rare malignancy, with an incidence of 1–2 cases per million population (1). Prognosis is generally poor due to its aggressive nature and tendency to recur (2). Nevertheless, there is significant heterogeneity in individual outcomes. The median overall survival (OS) of ACC patients is 3.2 years, with stage-specific median survival ranging from 24.1 years for patients with stage I ACC to 0.9 years for those with stage IV ACC (3). However, some patients with metastatic disease survived more than 10 years after diagnosis (4). Therefore, the establishment of prognostic factors to predict patient survival is important to aid clinical decision-making.

A previous literature review revealed that tumor stage, surgical margin status, presence of distant metastases, and tumor grade were significantly associated with prognosis (5). Complete surgical resection with negative margins affords the greatest opportunity for cure (3). However, advanced stage and positive remote organ metastases are associated with poor outcomes (2). Several studies have also proposed advanced age as a negative prognostic factor (6–8), although others have not found such a correlation (9, 10). Hypercortisolism has been reported to be negatively correlated with OS. The mechanism was possibly related to immunocompromised status but was still poorly understood (11, 12).

The aforementioned studies were mostly restricted to European and American populations. Prognostic investigations of ACCs in the Chinese population are limited. Only Dong et al. reported a survival benefit in a cohort of patients who underwent surgical resection at Peking, China (13). As such, the aim of our study was to assess the clinical characteristics of patients with ACC treated at a single tertiary center in Taiwan, and to investigate the prognostic implications of each factor on OS and progression-free survival.

## Methods

After the approval of research review board, the medical records of patients diagnosed with ACC at Taipei Veterans General Hospital between January 1992 and June 2021 were reviewed. Covariates included sex, age at diagnosis, presence of symptoms, tumor size, hormonal activity, tumor localization, surgical margins, European Network for the Study of Adrenal Tumours (ENSAT) stage, tumor grade, local invasion, metastatic status, large vessel invasion, and adjuvant therapy. Functional status of the tumor was determined through records of cortisol, androgen, and/or aldosterone hypersecretion before the operation. Surgical margins were assessed as follows: R0 resection was defined as the absence of residual disease; R1 resection was defined as a microscopically positive surgical margin; R2 resection was determined by macroscopic residual tumors; and RX resection indicated unknown status. Tumor grade was evaluated using the Ki-67 index, with Ki-67 ≤ 10% considered to be low-grade and Ki-67 > 10% considered to be high-grade (14).

The ENSAT classification system was adopted owing to its superiority in prognostic prediction compared with the International Union Against Cancer staging system (15). According to the ENSAT classification, stage I ACC was defined as a tumor measuring ≤ 5 cm in size without extra-adrenal invasion. Stage II ACC was a tumor > 5 cm but still confined to the adrenal gland. Stage III ACC was defined by the presence of local extension, vascular invasion, or lymphatic spread. Stage IV ACC was confirmed by positive distant metastases (16). OS was estimated from the date of diagnosis to the date of death. Progression-free survival was evaluated from the date of diagnosis to the date of documented progressive or recurrent disease.

### Statistical analysis

All statistical analyses were performed using SPSS version 25 (IBM Corporation, Armonk, NY, USA) to evaluate OS and progression-free survival according to the Kaplan-Meier method. Univariate Cox regression analysis was used to determine the association between each factor and OS and progression-free survival. Factors that were statistically significant (*p* < 0.05) were included in the multivariate Cox regression analysis to confirm independent prognostic influence. Quantitative statistics are expressed as mean and standard deviation (SD).

## Results

### Patient characteristics

A total of 67 patients were included in the study. Demographic information and basic characteristics of the patients are summarized in **Table 1**. None of the patients had hereditary malignancy syndromes. The mean age at diagnosis was 51.7 ± 16.1 years (median age, 50 years). More than one-quarter of the patients were diagnosed in their 40s, and 44 (66.7%) patients were female. The mean age of the male patients was 58.3 years, while the mean age of the female patients was 48.3 years (*p* = 0.015). However, the mean age did not differ between the different ENSAT stages. Nearly one-half of the patients (n = 33/67 [49.2%]) had stage IV disease. Among these patients, twenty-five were diagnosed with late presentation, while the other 8 had missed diagnosis initially. Only 5 (7.5%) were diagnosed with stage I ACC. Fifteen (22.4%) patients had stage II ACC, and 14 (20.9%) were diagnosed with stage III ACC. Thirteen patients (19.4%) had incidentally found tumor without any clinical manifestation. Local invasion was observed in 14 (20.9%) patients. Lymphatic metastasis was observed in 9 (13.4%) patients. The most common locations of distant metastases were the liver and lungs. Nineteen patients had liver metastases and 12 had lung metastases. Five patients presented with large vessel thrombosis. Twenty-four (35.8%) patients had functional ACC. Among these patients, the age at diagnosis was significantly lower than that of those with non-functional cancer (mean, 46.8 versus 55.9 years; *p* = 0.020). Twenty-one (*p* = 0.001) patients were female. Seventeen patients exhibited hypercortisolism, 6 had elevated androgen levels, and 2 were screened positively with primary aldosteronism (PA) based on aldosterone-to-renin ratio. One patient exhibited elevated aldosterone and cortisol levels. Among patients with cortisol excess, sixteen presented with overt Cushing syndrome including Cushingoid appearance, weight gain, irregular menstrual cycles and recently worsening glycemic status. The other one had subclinical form of hypercortisolism. Those who had hyperandrogenism complained of amenorrhea and hirsutism.

**Table 1:** Clinical characteristics of adrenocortical carcinoma patients.

Number of cases	67
Age	
<20	2(3.0%)
21-30	4(6.0%)
31-40	9(13.4%)
41-50	20(29.9%)
51-60	9(13.4%)
61-70	14(20.9%)
>70	9(13.4%)
Mean age at diagnosis(years)	51.7 ± 16.1
Tumor size	
≦5 cm	14(21.9%)
>5 cm	53(79.1%)
Male	23(34.3%)
Anatomic site	
Left	37(55.2%)
Right	28(41.8%)
Bilateral	2(3.0%)
ENSAT stage	
I	5(7.5%)
II	15(22.4%)
III	14(20.9%)
IV	33(49.2%)
Hormonally functioning tumor	24(35.8%)
Cortisol alone	16(23.9%)
Androgen alone	6(9.0%)
Aldosterone alone	1(1.5%)
Aldosterone and cortisol	1(1.5%)
Therapy received	
Surgery	51(76.1%)
Mitotane	49(73.1%)
Adjuvant chemotherapy	21(31.3%)
Adjuvant radiotherapy	8(11.9%)
R0 resection	12(17.9%)
Lymph node metastasis	9(13.4%)
Local invasion	14(20.9%)
Large vessel invasion	5(7.5%)
Clinical manifestation	
Symptomatic	45(67.2%)
Asymptomatic	22(32.8%)

### Surgical results and post-surgical treatment

All the patients were involved in multidisciplinary team care including urologists, endocrinologists, oncologists, radiotherapists, pharmacists, nurses and nutrition specialists. Fifty-one (76.1%) patients underwent surgery, almost all of whom underwent surgical resection at Taipei Veterans General Hospital, except for 2, who underwent surgery at other hospitals. Pathologists at the Taipei Veterans General Hospital reviewed the pathological slides of these 2 patients. Most patients underwent open surgery (n = 39/51 [76.5%]). R0 resections were recorded in 12 patients, among whom 8 were diagnosed with local disease (stage I + II), and 4 had stage III ACC.

Mitotane was administered to 49 (73.1%) patients, with a median dose of 1.5 g/day. The average duration of mitotane therapy was 40.9 months. The side effects recorded in our cohort included nausea, vomiting, diarrhea, skin rash, dizziness, lethargy, anorexia, hypothyroidism, gynecomastia, impotence, gait disturbance, slow response, hand tremor and hypertension, most of which were relieved by symptomatic treatment and down-titration of mitotane. The incidence of mitotane-related adrenal insufficiency was 38.8% (19/49), with mean onset of 5.3 months. Steroid supplements were administered to these patients. Clinical examination of serum mitotane levels is not available in Taiwan. Twenty-one patients underwent adjuvant chemotherapy, eighteen of which received etoposide, doxorubicin and cisplatin (EDP) regimen. Two patients underwent therapy with cisplatin and etoposide due to poor cardiac function, while the other one received therapy with cisplatin, etoposide and bleomycin. Eight patients underwent post-surgical radiotherapy. Palliative care teams were available to provide relief of symptoms related to cancer or treatment.

### Survival outcomes

**Figure 1:**
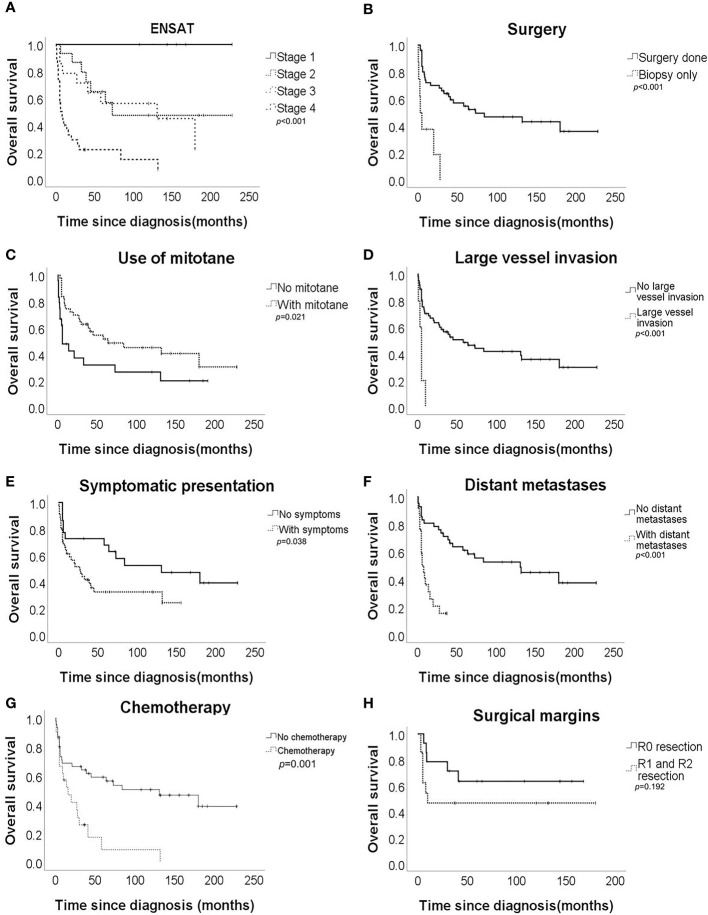
Overall survival of the adrenocortical carcinoma patients according to **(A)** European Network for the Study of Adrenal Tumours (ENSAT) stage, **(B)** surgical resection, **(C)** mitotane use, **(D)** large vessel invasion, **(E)** symptomatic presentation, **(F)** distant metastases, **(G)** chemotherapy, and **(H)** surgical margin.

Univariate analysis revealed that ENSAT stage, distant metastases, large vessel invasion, symptomatic presentation, use of mitotane, chemotherapy, and surgical therapy were significantly associated with OS (**Table 2** and **Figure 1**). Multivariate analysis (**Table 3**) revealed that ENSAT stage (hazard ratio [HR] 3.60 [95% confidence interval (CI) 1.25–10.38]), and great vessel invasion (HR 5.19 [95% CI 1.75-15.37]) were factors negatively and independently associated with OS. In contrast, administration of mitotane was associated with better outcomes (HR 0.27 [95% CI 0.11–0.70]). The mean OS was 160.8, 85.5, 81.8 and 20.3 months for stage I, II, III, and IV patients, respectively. The mean OS across all stages was 58.2 months. The 5-year OS percentage was 35.8%.

**Table 2:** Univariate Cox regression analysis on overall survival.

Variable	Hazard ratio(95% CI)	P
Sex (Female vs. male)	1.11(0.59-2.11)	0.741
Age (years)	1.01(0.99-1.03)	0.483
ENSAT stage (III+IV vs.I+II)	3.86(1.70-8.80)	0.001
Tumor size (>5cm vs.≦5cm)	2.29(0.71-7.45)	0.168
DHEA-S level (>20 μmol/L vs.≦20 μmol/L)	0.45(0.10-2.06)	0.302
Lymph node metastasis (Presence vs. absence)	1.58(0.69-3.62)	0.275
Distant metastasis (Presence vs. absence)	4.97(2.34-10.57)	<0.001
Large vessel invasion (Presence vs. absence)	5.48(2.00-15.01)	0.001
Metastatic organs (Lung vs. liver)	2.41(0.92-6.32)	0.073
Metastatic organs (Lung+liver vs. liver alone)	1.88(0.39-9.04)	0.433
Local invasion (Presence vs. absence)	2.36(0.90-6.18)	0.080
Surgical therapy (No vs. yes)	2.08(1.42-3.05)	0.001
Surgical type (Laparoscopic vs. open)	1.55(0.57-4.18)	0.390
R0 resection (No vs. yes)	1.86(0.59-5.90)	0.290
Ki-67 (>10% vs. ≦10%)	1.62(0.18-14.55)	0.666
Use of mitotane (Yes vs. no)	0.49(0.26-0.92)	0.026
Chemotherapy (Yes vs. no)	2.87(1.49-5.54)	0.002
Functional activity (Presence vs. absence)	1.12(0.61-2.08)	0.712
Cortisol hypersecretion (Presence vs. absence)	1.43(0.75-2.78)	0.280
Symptomatic presentation (Yes vs. no)	2.07(1.02-4.20	0.044

**Table 3:** Multivariate Cox regression analysis on overall survival.

Variable	Hazard ratio(95% CI)	P
ENSAT stage (III+IV vs.I+II)	3.60(1.25-10.38)	0.018
Distant metastasis (Presence vs. absence)	1.94(0.81-4.65)	0.137
Large vessel invasion (Presence vs. absence)	5.19(1.75-15.37)	0.003
Surgical therapy (No vs. yes)	1.128(0.65-1.94)	0.685
Use of mitotane (Yes vs. no)	0.27(0.11-0.70)	0.007
Chemotherapy (Yes vs. no)	1.26(0.57-2.79)	0.568
Symptomatic presentation (Yes vs. no)	1.74(0.76-3.98)	0.190

In terms of progression-free survival, univariate analysis revealed that ENSAT stage, distant metastases, large vessel invasion, surgical therapy and chemotherapy had a statistically significant impact (**Table 4**). However, no single factor was associated with progression-free survival. The mean progression-free survival of the entire cohort was 39.0 months, while stage-specific progression-free survival was 160.8, 58.7, 52.6, and 5.8 months in patients with stage I, II, III, and IV disease, respectively. The 5-year progression-free survival was 25.4%.

Table 4Univariate and Multivariate Cox regression analysis on progression-free survival.

**Table:** 

Variables of univariate Cox regression analysis	Hazard ratio(95% CI)	P
Sex (Female vs. male)	0.89(0.47-1.68)	0.716
Age (years)	1.01(0.99-1.03)	0.336
ENSAT stage (III+IV vs.I+II)	3.90(1.70-8.94)	0.001
Tumor size (>5cm vs.≦5cm)	2.46(0.75-8.00)	0.136
DHEA-S level (>20 μmol/L vs.≦20 μmol/L)	0.41(0.09-1.83)	0.245
Local invasion (Presence vs. absence)	2.17(0.83-5.65)	0.112
Lymph node metastases (Presence vs. absence)	1.64(0.73-3.73)	0.234
Distant metastasis (Presence vs. absence)	4.43(2.16-9.07)	< 0.001
Large vessel invasion (Presence vs. absence)	3.38(1.27-8.98)	0.014
Metastatic organs (Lung vs. liver)	1.33(0.47-3.80)	0.592
Surgical therapy (No vs. yes)	4.96(2.04-12.05)	< 0.001
Surgical type (Laparoscopic vs. open)	1.61(0.60-4.31)	0.348
Ki-67 (>10% vs. ≦10%)	2.54(0.28-23.08)	0.409
R0 resection (No vs. yes)	1.84(0.58-5.87)	0.301
Use of mitotane (Yes vs. no)	0.53(0.28-1.00)	0.050
Chemotherapy (Yes vs. no)	2.87(1.50-5.49)	0.001
Functional activity (Presence vs. absence)	1.14(0.61-2.10)	0.683
Cortisol hypersecretion (Presence vs. absence)	1.40(0.74-2.65)	0.303
Symptomatic presentation (Yes vs. no)	1.82(0.91-3.62)	0.090

**Table:** 

Variables of multivariate Cox regression analysis	Hazard ratio(95% CI)	P
ENSAT stage (III+IV vs.I+II)	2.34(0.92-5.94)	0.074
Distant metastasis (Presence vs. absence)	2.28(0.99-5.24)	0.052
Large vessel invasion (Presence vs. absence)	1.87(0.68-5.15)	0.227
Surgical therapy (No vs. yes)	1.38(0.90-2.10)	0.139
Chemotherapy (Yes vs. no)	1.19(0.55-2.59)	0.662

Regarding survival among different ENSAT stages, there was still better OS for patients with stage III than for those with stage IV ACC (HR 0.30 [95% CI 0.13–0.70]). On the other hand, there was no difference in OS between those with stage II and stage III disease (HR 1.27 [95% CI 0.46–3.52]) or even between stage I+II and stage III ACC (HR 1.89 [95% CI 0.69–5.23]). Furthermore, there was no difference in progression-free survival between those with stage II and stage III ACC (HR 1.22 [95% CI 0.44–3.36]).

Regarding the effects of mitotane on clinical outcomes, the difference in OS was most apparent in patients with stage IV disease. The mean OS of patients with stage IV disease who underwent mitotane therapy (n = 18/33 [54.6%]) was 33.5 months, while those who did not receive mitotane (n = 15/33 [45.4%]) only survived 4.4 months on average (*p* = 0.010). Furthermore, mitotane therapy had a borderline beneficial effect on progression-free survival (HR 0.53 [95% CI 0.28–1.00]). Subgroup analysis revealed that the use of mitotane also resulted in better progression-free survival of patients with stage IV ACC (HR 0.39 [95% CI 0.17–0.91]). Moreover, the functional status of tumor did not affect the association between mitotane therapy and improved OS (HR 0.74 [95% CI 0.25-2.22]) and progression-free survival (HR 0.42 [95% CI 0.14-1.36]). However, mitotane administration to patients with stage I-III disease did not result in better survival.

## Discussion

Results of our study provide insights into the prognostic predictors of ACC in a sample of ethnic Taiwanese population. The median overall survival of our cohort was similar to the literature which ranged from 15-44% (3). ENSAT stage had the greatest impact on OS. Significant differences in clinical outcomes were more pronounced in those with more advanced stages of disease. No difference in survival was observed between patients with stage II and III ACC. There have been inconsistent results regarding survival between stage II and stage III disease. Luton et al. confirmed that regional disease did not influence survival (6), whereas Icard et al. reported worse outcomes in patients with stage III ACC than in those with stage I+II ACC (17). Surgery remains the sole cure for ACC. Patients with stage I or II ACC have the best chance of complete tumor removal due to localized disease. However, tumors with invasion to adjacent tissues or lymph nodes can still be completely removed by experienced surgeons through meticulous resection (14). Open en bloc surgery was performed in most patients in our study. The open procedure is recommended by current practice guidelines owing to more extensive clearance of tumors and comprehensive sampling of lymph nodes (18). Lymphadenectomy performed during surgery has also been reported to improve survival (19). Moreover, centers with expertise in ACC have a more positive influence on clinical outcomes (20, 21). Care by multidisciplinary team evaluation was adopted in our cohort. Multidisciplinary care has been shown to improve overall and progression-free survival in patients with stage III-IV ACC (22). Similar survival benefits were also demonstrated in tumor stage I-III in a dutch study which revealed surgery performed outside the hospitals integrating care from multiple expertise was associated with increased risks of death (HR 1.96 [95% CI 1.01–3.81], *p*=0.047) *(* 20). These facts may explain the good prognosis observed in patients with stage III ACC in our series. Additionally, patients with stage IV ACC are susceptible to disease progression. This may explain why patients with stage IV disease experience worse OS than those with stage III ACC. Nevertheless, the number of patients with local or regional ACC in our cohort was small. Given the various surgical methods, there was no difference in OS between patients who underwent open surgery and laparoscopic surgery in our study. A meta-analysis including 9 retrospective case-control studies revealed similar cancer-specific mortality rates between laparoscopic adrenalectomy, adopted mostly for patients with smaller tumors, and open adrenalectomy. However, increased peritoneal metastasis is more often reported in patients who underwent laparoscopic surgery (23). No prospective randomized trial has directly compared these two surgical methods.

Our cohort exhibited obvious survival benefits with mitotane administration among those with stage IV ACC. Mitotane is a derivative of dichlorodiphenyltrichloroethane (DDT) (14). Its active metabolites induce adrenal cell necrosis by blocking mitochondrial respiratory chain complexes I and IV, and disrupting the mitochondrial membrane (24). Mitotane also inhibits sterol-*O*-acyl transferase 1 (SOAT1), which contributes to the accumulation of free fatty acids and cholesterol. These redundant materials cause stress in the endoreticulum and activate the intrinsic apoptotic pathway (24). Initially, mitotane was administered to patients with advanced ACC, with a response rate ranging from 5–49% (25). These observations established the rationale for adjuvant mitotane therapy in patients at high risk for recurrent or metastatic disease. However, in these studies, mitotane was usually combined with chemotherapy. It is difficult to determine whether the therapeutic benefits result from mitotane alone. In a meta-analysis including 6 retrospective studies, mitotane therapy resulted in improved mortality but not in decreased recurrence (18). Treatment options based on predicted prognosis may have confounding effects in these studies, which can be hardly corrected. Although current guidelines recommend adjuvant mitotane therapy for patients with stage III-IV ACC or any stage with a Ki-67 index >10%, who do not have macroscopic residual disease after surgery (18), this recommendation is based on low-quality evidence and expert opinions. Moreover, the randomized ADIUVO trial reported no additional benefits of adjuvant mitotane in recurrence-free survival and OS among patients with stage I-III ACC, R0 surgery, and Ki-67 ≤10% compared with observation-alone group, which was similar to our finding that mitotane did not add survival benefits in stage I-III disease (26). Well-designed prospective randomized trials are still needed to confirm the efficacy of mitotane in more advanced ACC. On the other hand, higher concentration of mitotane above 14 mg/L had independently positive impact on survival if mitotane is administered (27). The time needed to reach this target range was also significantly associated with disease recurrence. Furthermore, longer period in target concentration during maintenance phase of mitotane therapy resulted in reduced recurrence (HR 0.93 [95% CI 0.88–0.98], *p*<0.01) *(* 28). Last but not least, patients with advanced disease and use of mitotane often had multiple physical and mental illness. Early introduction of palliative care along with standard treatment improved quality of life, survival and psychological burden of both patients and caregivers (29).

Moreover, our study revealed a negative association between large-vessel invasion and OS, which is seldom discussed with regard to prognostic prediction in most studies. In our series, these patients usually had late-stage disease and positive surgical margins. The mean OS was only 4.8 months. Turbendian et al. reported a decrease in 3-year OS from 93% to 29% and in 3-year recurrence-free survival from 67% to 15% in patients with large-vessel invasion (30). An association between adrenal hormone hypersecretion and symptomatic clinical manifestations with large-vessel thrombosis was also found in that study. Although venous thrombosis can be removed using sophisticated techniques, Chiche et al. found that perioperative mortality in patients with inferior vena cava invasion was approximately 10–15%. The long-term survival of these patients was poor owing to the association with metastases and lag in correct diagnosis (31). However, the association between vascular invasion and survival may be confounded by advanced stage and multiple distant metastases.

Less patients presented with hormonal excess (35.8%) in our cohort compared with most series in which 45-70% of patients had active hormone production (2). The majority of patients (49.2%) in our study were diagnosed with stage IV disease, which was still true for patients diagnosed before and after 2010. However, with increased availability of advanced imaging techniques, more and more patients were diagnosed with stage II ACC recently (32). Regarding other prognostic factors, our cohort did not reveal significant association of hypercortisolism, advanced pathologic features or status of surgical margin with overall survival.

Our study had some limitations, among which included its single-center, retrospective design and inherent selection bias; moreover, the size of the cohort was relatively small. Referral bias may have also occurred because adrenocortical carcinoma is rare. The clinical presentations may have been altered by previous failed treatments at other hospitals. Furthermore, details including Eastern Cooperative Oncology Group (ECOG) performance status of the patients and mitotic index could not be traced due to missing records or incomplete documents. Assay for detection of mitotane level was also not available. The confirmation tests of PA were not performed at the diagnosis of ACC. All cases were only screened positively with PA based on aldosterone-to-renin ratio. However, the strengths of our study are that it analyzed prognostic predictors of patients with ACC in a sample of the ethnic Taiwanese population and that baseline characteristics were similar to those of the global population. A multicenter prospective cohort study, with complete records of patient characteristics and tumor grades, is needed to better stratify the prognostic factors of ACC in Taiwan.

## Conclusion

Advanced ENSAT stage and large vessel invasion were negatively associated with OS, whereas the use of mitotane improved clinical outcomes. However, no single factor had an independent influence on progression-free survival.

## Data availability statement

The data analyzed in this study is subject to the following licenses/restrictions: Paper dataset. Requests to access these datasets should be directed to Liang-Yu Lin, tristan074@gmail.com.

## Ethics statement

The studies involving human participants were reviewed and approved by Institutional review board, Taipei Veterans General Hospital. Written informed consent for participation was not required for this study in accordance with the national legislation and the institutional requirements.

## Author contributions

L-HP, and L-YL were the main conductors of this study and contributed to the study conception and design, implementation, statistical analysis, interpretation, the preparation and finalization of the manuscript. C-CY contributed to the study conception and design, implementation, and the preparation of the manuscript. C-JH and X-NN contributed to the study conception and design, and data collection. All authors contributed to the article and approved the submitted version.
